# The ORVAC trial: a phase IV, double-blind, randomised, placebo-controlled clinical trial of a third scheduled dose of Rotarix rotavirus vaccine in Australian Indigenous infants to improve protection against gastroenteritis: a statistical analysis plan

**DOI:** 10.1186/s13063-020-04602-w

**Published:** 2020-08-26

**Authors:** Mark A Jones, Todd Graves, Bianca Middleton, James Totterdell, Thomas L Snelling, Julie A Marsh

**Affiliations:** 1 Wesfarmers Centre of Vaccines and Infectious Diseases, Telethon Kids InstituteUniversity of Western Australia, Perth, 6009 WA Australia; 2 Perth Children’s Hospital, 15 Hospital Ave, Perth, 6009 WA Australia; 3 Curtin University, School of Public Health, Perth, WA Australia; 4 Berry Consultants, 3345 Bee Caves Rd Suite 201, Austin, 78746 TX USA; 5 Menzies School of Health Research, Royal Darwin Hospital Campus, Rocklands Drive, Casuarina, 0811 NT Australia

**Keywords:** Statistical methods, Adaptive design, Interim analysis, Randomised controlled trial, Bayesian, Infectious disease, Rotavirus vaccine, Rotarix, RV1

## Abstract

**Objective:**

The purpose of this double-blind, randomised, placebo-controlled, adaptive design trial with frequent interim analyses is to determine if Australian Indigenous children, who receive an additional (third) dose of human rotavirus vaccine (Rotarix, GlaxoSmithKline) for children aged 6 to < 12 months, would improve protection against clinically significant all-cause gastroenteritis.

**Participants:**

Up to 1000 Australian Aboriginal and Torres Strait Islander (hereafter Indigenous) infants aged 6 to < 12 months will be recruited from all regions of the Northern Territory.

**Interventions:**

The intervention is the addition of a third scheduled dose of human monovalent rotavirus vaccine.

**Co-primary and secondary outcome measures:**

ORVAC has two co-primary outcomes: (1) anti-rotavirus IgA seroconversion, defined as serum anti-rotavirus IgA ≥ 20 U/ml 28 to 55 days post Rotarix/placebo, and (2) time from randomisation to medical attendance for which the primary reason for presentation is acute gastroenteritis or acute diarrhoea illness before age 36 months. Secondary outcomes include (1) change in anti-rotavirus IgA log titre, (2) time from randomisation to hospitalisation with primary admission code presumed or confirmed acute diarrhoea illness before age 36 months, (3) time from randomisation to hospitalisation for which the admission is rotavirus confirmed diarrhoea illness before age 36 months and (4) time from randomisation to rotavirus infection (not necessarily requiring hospitalisation) meeting the jurisdictional definition before age 36 months.

**Discussion:**

A detailed, prospective statistical analysis plan is presented for this Bayesian adaptive design. The plan was written by the trial statistician and details the study design, pre-specified adaptative elements, decision thresholds, statistical methods and the simulations used to evaluate the operating characteristics of the trial. As at August 2020, four interim analyses have been run, but no stopping rules have been triggered. Application of this SAP will minimise bias and supports transparent and reproducible research.

**Trial registration:**

Clinicaltrials.gov NCT02941107. Registered on 21 October 2016

**Original protocol for the study:**

10.1136/bmjopen-2019-032549

## Background

Despite the introduction of rotavirus vaccine into the childhood vaccination schedule in 2006, Northern Territory Indigenous children remain more than 20 times more likely to be hospitalised with rotavirus gastroenteritis than non-Indigenous children in other Australian states and territories [1]. Data from remote communities in the Northern Territory suggests that 77% of children have at least one documented episode of clinic attendance for diarrhoea before their first birthday, with a median of three (IQR 1–5) clinical presentations for diarrhoea per child in the first year of life [2].

The current vaccination schedule with Rotarix is at 2 and then 4 months of age. We hypothesise that the routine addition of a third scheduled dose of Rotarix for NT Indigenous infants, administered between 6 months and less than 12 months old, will improve protection against clinically important rotavirus gastroenteritis.

This statistical analysis plan (SAP) provides a priori specification of the decision-making rules and the statistical methods to be used. It is intended to disseminate practical knowledge on adaptive trials to trialists that are new to these designs. The SAP was prepared after data collection had commenced, but prior to observing any of the data. The coordinating principal investigator (TLS) was responsible for approving and signing off the SAP, and the document has also been reviewed and approved by an independent data monitoring safety board (DMSB). The SAP is consistent with the CONSORT 2010 Statement [3] and further guidelines [4–6] and supports transparent and reproducible research.

As at August 2020, four interim analyses have been run, but no stopping rules have been triggered.

## Adaptive designs

While fixed design clinical trials are conceptually straightforward, they suffer from rigidity and frequently end with inconclusive results [7, 8]. Contrastingly, adaptive designs (ADs) allow for pre-specified adaptations that modify the design as the trial progresses and as data is accumulated [9].

Typical adaptations include dynamic changes to sample size, dropping treatment arms for futility, response adaptive randomisation, seamless phase II/III trial transitions, study population enrichment and early stopping rules [10] that are triggered under predefined conditions [11]. Consequently, ADs may be completed sooner, cost less to run, reduce the number of patients exposed to inferior treatments and provide more clinically relevant data than fixed designs [12]. However, ADs are not without their own limitations. The processes associated with obtaining funding, planning and designing adaptive trials are complex and can require more effort and time than traditional trials [8]. Furthermore, developing simulations [13] to explore the frequentist operating characteristics (e.g. type I error and power) requires specialist staff and/or consulting services, custom software infrastructure and high-performance computing facilities [7].

Historically, ADs and platform trials (a variant of an adaptive trial) have generally been implemented in phase I and II settings in the USA, with many originating from the University of Texas MD Anderson Cancer Center [11, 14, 15]. However, interest in ADs is growing and they are now being deployed and evaluated in phase III (e.g. [16–18]) and IV settings [9, 19]. In early 2016, the REMAP-CAP (Randomised, Embedded, Multifactorial Adaptive Platform trial for Community-Acquired Pneumonia) trial, an embedded platform trial run jointly in Europe, Australia and New Zealand, commenced. REMAP-CAP has served as a catalyst, motivating Australian trialists to raise funds, develop capacity and become involved in adaptive trials to answer their research questions. Examples include BEAT CF [20] and GBM Agile [21]. The ORVAC trial is one of several Bayesian ADs that are in development at the Telethon Kids Institute in collaboration with Berry Consultants.

## ORVAC study design

ORVAC is a pragmatic, investigator-led, double-blind, randomised, placebo-controlled Bayesian adaptive clinical trial testing a third scheduled dose of Rotarix rotavirus vaccine (versus usual care) in Australian Indigenous infants to improve protection against clinically important gastroenteritis. It has the following key features:

- Double-blind, randomised, placebo-controlled trial (neither the outcome observer nor participant’s caregivers know the treatment status);
- Non-fixed sample size up to 1000 participants (up to the first 250 with venous sampling) based on Bayesian stopping rules (minimum sample size of 70 for predicting futility);
- Fixed 1:1 parallel group enrolment into the active and control arm throughout the trial;
- Frequent interim analyses;
- Evaluation of intervention effects in the Darwin urban region compared to remote/very remote regions; and
- Study participation is from randomisation until the end of follow-up at 36 months of age.

### Adaptive elements

The adaptive elements all relate to sample size. Enrolment will continue up to the maximum sample size unless one of the following criteria for a statistical trigger is met at an interim analysis:

1. Stop venous sampling because the treatment arm shows overwhelming evidence of an increase in seroconversion.
2. Stop for futility, ceasing the trial before the maximum sample size is reached because the probability of observing a beneficial treatment effect is very small, even if the trial were continued to its maximum sample size.
3. Stop for expected success, ceasing the trial before the maximum sample size is reached because:
  - Futility in both the immunological and clinical outcome is very unlikely; and
  - The treatment group shows overwhelming evidence of an increase in the median time to medical attendance for which the primary reason for presentation is presumed or confirmed acute gastroenteritis or acute diarrhoea illness.

Futility and expected success are tested sequentially, which implies that if we establish futility in either the immunological or clinical outcome, then we will not test for expected success.

## Trial population

ORVAC has two population subgroups: (1) children who normally reside in a major city, an inner or outer regional area and (2) children residing in remote or very remote areas according to the Australian Government Department of Health Australian Standard Geographical Classification-Remoteness Area (ASGC-RA) system. While baseline characteristics for immunological status and median time to medical attendance/hospitalisation are not well understood for this population, comparable settings have an RV vaccine efficacy of around 50%. However, in the NT Indigenous population, protection has been noted to wane after the first year of life, estimated to fall to a rate of only 10% [22]. Estimates for the rate of hospitalisations due to acute gastroenteritis are also highly variable with median times to hospitalisations reported to be between 15 and 40 months from birth [1].

The clinical data from this trial will be analysed and reported on an intention-to-treat (ITT) basis with all randomised participants contributing to the analysis of the co-primary endpoints. Specifically, for the ITT analysis:

- Patients will be analysed in the group they were allocated to;
- Patients not receiving Rotarix/placebo will be retained;
- False inclusions will be retained;
- Protocol deviations will not result in exclusion; and
- The potential effect of missing values will be examined (see later).

We will also produce companion analyses on a per-protocol (PP) basis using the subset of participants that completed without protocol violation.

## CONSORT diagram

We will prepare an expanded CONSORT diagram suitable for a parallel two-armed adaptive trial. We will record the start and end dates of accrual, the flow of participants through the study including the completeness of follow-up as per the CONSORT statement [23]. We will note participants enrolled and randomised and eligibility for analyses.

## Randomisation

Stratified (regional vs remote), random allocation of two treatment arms to contiguous randomisation numbers (1 to 1000) was provided by JM. The allocation was computer-generated using random permuted block sizes between 6 and 20. The allocation ratio within these strata is 1:1, and MAJ maintains the password-protected file that contains the allocation sequences.

## Blinding

The ORVAC trial is double-blind with neither the participants nor the research staff having knowledge to patient treatment status. MAJ is solely responsible for interim analyses and reporting to the Data Safety Monitoring Board (DSMB). JM is responsible for quality control review on the interim analysis reports, with both MAJ and JM being unblinded. JT is responsible for code review, but remains blinded to the randomisation and results. All other staff and investigators are blinded. Interim analyses are discussed in closed session between MAJ and the DSMB with no investigators present. After each interim analysis is completed and approved by the DSMB, a recommendation for continuing the study (with no reference to the results) is reported to TS by the DSMB. All randomisation lists and analyses are kept on a secure server for which only MAJ has access.

## Study objectives and outcomes

The purpose of this study is to determine if Indigenous children who receive an additional dose of Rotarix between the ages of 6 and 12 months will have an increased anti-rotavirus serum IgA seroconversion and an increase in the time to medical attendance due to gastroenteritis in the first 3 years of life, compared to those who receive placebo.

### Co-primary

The primary objective is quantified through a clinical and an immunological outcome designed to measure the clinical effectiveness of an additional scheduled dose and any change in the immune response.

The immunological outcome is anti-rotavirus IgA seroconversion, defined as serum anti-rotavirus IgA ≥ 20 U/ml at 28 to 55 days post Rotarix/placebo among infants with anti-rotavirus serum IgA < 20 U/ml prior to administering the third dose.

The clinical outcome is the time from randomisation to first medical attendance (hospitalisation, emergency department presentation, medical clinic presentation) for which the primary reason for presentation is presumed or confirmed all-cause acute gastroenteritis or acute diarrhoea illness between randomisation and age 36 months.

### Secondary

The secondary objectives are exploratory and examine other aspects of effectiveness and immune response, see below and Table 1. Additionally, we will report on the safety and tolerability by examining the occurrence of intussusception potentially attributable to the intervention and the occurrence of serious adverse events.

**Table 1:** Analysis methods for secondary outcomes

Endpoint	Overview
Time from randomisation to hospitalisation for which the primary coded reason for admission is presumed or confirmed acute gastroenteritis or acute diarrhoea illness between randomisation and age 36 months.	Summary of the median and inter-quartile range for each treatment arm. The analysis will follow the form of the analysis for the primary clinical endpoint. We will provide a competing risk analysis as discussed in the main text.
Time from randomisation to hospitalisation for which rotavirus confirmed diarrhoea illness occurs between randomisation and age 36 months.	Summary of the median and inter-quartile range for each treatment arm. The analysis will follow the form of the analysis for the primary clinical endpoint. We will provide a competing risk analysis as discussed in the main text.
Time from randomisation to rotavirus infection meeting the jurisdictional case definition between randomisation and age 36 months.	Summary of the median and inter-quartile range for each treatment arm. The analysis will follow the form of the analysis for the primary clinical endpoint. We will provide a competing risk analysis as discussed in the main text.
Change in anti-rotavirus IgA log titre between administration of intervention (RV1/placebo) and 28 to 55 days post dose.	We will adopt a robust linear regression analysis assuming the errors follow *t* distribution with between 3 and 7 degrees of freedom.
Frequency of intussusception fulfilling Brighton criteria within the first 28 days after administration of the third dose	Descriptive summary.
Frequency of serious adverse events between randomisation and age 36 months.	Descriptive summary.

Clinical outcomes comprise:

- Time from randomisation to hospitalisation for which the primary coded reason for admission is presumed or confirmed acute gastroenteritis or acute diarrhoea illness before age 36 months;
- Time from randomisation to hospitalisation for which the primary reason for admission is rotavirus-confirmed diarrhoea illness before age 36 months; and
- Time from randomisation to rotavirus infection (not necessarily requiring hospitalisation) that meets the jurisdictional case definition (for disease notification) before age 36 months.

Immunological outcomes comprise:

- Change in anti-rotavirus IgA log titre between administration of the Rotarix or placebo dose and 28 to 55 days post dose.

Safety outcomes comprise:

- The occurrence of intussusception fulfilling Brighton criteria [24] within the first 28 days after administration of the Rotarix or placebo dose; and
- Serious adverse events as defined by [25] between randomisation and age 36 months.

### Competing risks

Conventional statistical methods for survival analysis assume independent or noninformative censoring. However, the presence of competing risks violates the noninformative censoring assumption because the occurrence of one event influences the likelihood of a competing event from occurring [26]. While the co-primary time-to-event outcome defined here does not compete with any other event, the secondary clinical time-to-event outcomes and safety outcomes may represent competing risks. This is true even though the secondary clinical outcomes are not strictly mutually exclusive as it is sufficient that the occurrence of one event influences the probability of subsequent events of a different type for the competing risks context to be relevant.

Competing risks require special handling, and we return to this topic in a later section.

## Statistical analyses

ORVAC is a superiority trial that uses Bayesian methods for inference and decision-making. Unless otherwise noted, all parameter estimates will be reported as means or medians with 95% credible intervals.

### Descriptive statistics

Participant characteristics will be summarised by treatment group and stratified by locality. No formal statistical testing will be performed to compare groups at this stage. We will provide quantitative summaries of the participant data including:

- Number of participants in ITT;
- Age at randomisation;
- Number of prior doses of Rotarix vaccination;
- Sex;
- Common comorbidities at randomisation;
- Breast feeding status;
- Anthropometric indices;
- Location of residence (urban versus remote);
- Proportion seropositive at baseline and seropositive/seroconverted at follow-up;
- Change in anti-rotavirus IgA between administration and second follow-up;
- Frequency of medical attendance events;
- Time to medical attendance events;
- Frequency of censoring;
- Frequency of intussusception;
- Frequency of adverse events; and
- Occurrence and timing of gastroenteritis outbreaks by community.

### Analysis of co-primary outcomes

We will assess both the immunological and clinical outcomes in a Bayesian framework with all model results reported. All models will be fit using Markov Chain Monte Carlo (MCMC).

#### Immunological co-primary outcome

The immunological outcome will be modelled using logistic regression including a covariate for treatment status (control arm coded as 0, treatment coded as 1). Denoting *y*_*i*_ as the seroconversion status, *π*_*i*_ as the probability of seroconversion and trt_*i*_ as an indicator variable for group membership for individual *i*, in the simplest case, we have:

1 $$\begin{array}{*{20}l} y_{i} &\sim \text{Bin}(1, \pi_{i}) \end{array} $$

2 $$\begin{array}{*{20}l} \text{logit}(\pi_{i}) &= \beta_{1} + \beta_{\text{trt}} \times \text{trt}_{i} \end{array} $$

However, we will also fit additional models that adjust for locality (urban versus remote), locality by treatment interaction, sex of participant, breast-feeding status in the 7 days prior to enrolment and community-specific indicators for gastroenteritis outbreaks.

We will use independent Student *t* distribution priors with location zero and scale 3 with 7 degrees of freedom that are recommended for general purposes [27, 28]. These priors imply

- The parameters are as likely to be positive as they are to be negative;
- The intercept is consistent with baseline log-odds between − 10 and 10; and
- A unit change in any covariate would be unlikely to exceed an absolute change of 5 on the log-odds scale.

We note that these priors are similar to normal priors, which are also suitable for logistic regression, but the Student *t* has slightly heavier tails [28]. The Student *t* priors are considered weakly informative and produce stable, moderately regularised and robust estimates. When using the Student *t* priors, it is recommended that binary independent variables are shifted to have a mean of zero and differ by one and that continuous independent variables have a mean of zero and a standard deviation of 0.5 [27]. Therefore, we will adopt this transformation, which puts all the input variables onto the same scale.

We will calculate the probability that the log odds ratio (*β*_trt_) is greater than zero. If this probability exceeds 0.97, chosen by simulation to control the type I error, we will conclude a successful treatment effect of increased probability of seroconversion in the treatment arm. In notation, we conclude a treatment effect has been demonstrated if $\mathbb {P}(\beta _{\text {trt}} > 0)> 0.97$. We will report both absolute values of the proportion of participants that seroconverted in each arm and the treatment effect size as a difference in proportions and as odds ratios.

#### Clinical co-primary outcome

In time-to-event analyses, the Cox proportional hazards (PH) semi-parametric model [29] is commonly applied. This model does not require knowledge of the baseline hazard function, which is generally held as its chief advantage. However, parametric models (and some semi-parametric variants) have advantages such as greater efficiency, they provide smoothly estimated survival functions and are easy to fit [30–34]. For ORVAC, we will adopt a Weibull proportional hazard (PH) model with covariates introduced through the scale parameter [34]. We will undertake model checking using standard methods of posterior predictive checks, leave-one-out cross-validation and information criterion [34–37].

For the Weibull PH model, denoting the time to event as *t*_*i*_ for individual *i*, we have:

3 $$\begin{array}{*{20}l} t_{i} &\sim \mathrm{W}(\lambda_{i}, a) \end{array} $$

4 $$\begin{array}{*{20}l} h(t_{i}) &= \lambda^{*} a t_{i}^{a-1} \end{array} $$

where *h*(*t*_*i*_) is the hazard function. In the simplest case, we will set the scale parameter to *λ*^∗^=*λ*exp(*θ*_trt_×trt_*i*_), with the absence of an intercept being intentional [37], yielding a PH model with shape parameter *a* and with both *λ*>0 and *a* such that the hazard increases when *a*>1 and decreases when *a*<1. The corresponding survival function of the Weibull model is:

5 $$\begin{array}{*{20}l} S(t_{i}) = \text{exp}(-\text{exp}(\theta_{trt} \times \text{trt}_{i}) \lambda t^{a}) \end{array} $$

from which it is implied that a plot of log(−log*S*(*t*_*i*_)) versus log(*t*_*i*_) will be approximately linear if the Weibull distributional assumption is reasonable. Furthermore, if analogous plots constructed for each treatment group yield parallel lines, then the proportional hazard assumption is valid. We will undertake model checking via the above heuristics and the usual Bayesian methods of posterior predictive checks and information criterion-based assessments. Finally, in cases where the PH assumption is violated, we will consider introducing time-dependent covariates or constructing an accelerated failure time (AFT) formulation as alternative strategies [38, 39].

For the shape parameter, *a*, we will adopt an exponential distribution prior with rate 0.7. This is consistent with values less than 10 and supports increasing and decreasing hazards with approximately equal probability. For the parameters in the linear predictor (the scale parameter and the hazard ratios), we will adopt independent normal priors (mean zero and standard deviation of 10). The normal priors are consistent with values on the log scale between − 15 and 15.

We will report the median time to event in each group and hazard ratios to quantify the treatment effect. In an analogous approach to that used in the immunological outcome, we will compute the probability that the parameter estimate for the treatment term is less than 0. If this probability exceeds 0.97 in the covariate-adjusted model, then we will conclude that a third scheduled Rotarix dose results in a treatment effect corresponding to a lower hazard of medical attendance in the treatment arm. In notation, we will conclude a treatment effect if $\mathbb {P}(\theta _{trt} < 0)> 0.97$.

We will use the methods as described above in the interim analyses including a single covariate for the treatment effect and using the predictive probability thresholds summarised in Table 2 for decision-making.

**Table 2:** Probability thresholds for evaluating statistical triggers at interim and final analyses

Posterior/predictive	Decision	Threshold	Comment
Posterior	Win	0.97	Probability threshold to test that treatment difference is greater than zero
Predictive	Expected success	0.90	Proportion of successful trials must be greater than this threshold to claim expected success
Predictive	Futility	0.05	Proportion of successful trials must be in less than this threshold to claim futility
Predictive	Stop venous sampling	0.90	Proportion of successful trials must be greater than this threshold to stop venous sampling

In the final analyses for the clinical endpoint, if $\mathbb {P}(\theta _{trt} < 0) > 0.97$ in the adjusted model, we will claim trial success.

### Analyses of secondary outcomes

The secondary endpoints comprise time-to-event measures, discrete measures and continuous measures. Short descriptions of the methods to be used for each secondary outcome are detailed in Table 1.

As noted earlier, conventional statistical methods for the analysis of survival data assume that competing risks are absent. Given that the clinical outcomes (and adverse and serious adverse events) represent competing risks, the results from a sub-distributional hazard model will also be reported as has been recommended for RCTs [40, 41].

### Interim analyses of co-primary outcomes

Bayesian adaptive trials rely on accumulating data and pre-specified decision rules to trigger adaptations. However, in order to ensure trial integrity, extensive simulation of the trial is required to investigate and quantify the operating characteristics such as the type I error rates, power and expected sample size under a range of hypothetical trial scenarios.

In this section, we outline the decision processes used for the interim analyses. Details on the simulations that were used to evaluate the operating characteristics of the trial are provided in a later section.

**Fig. 1:**
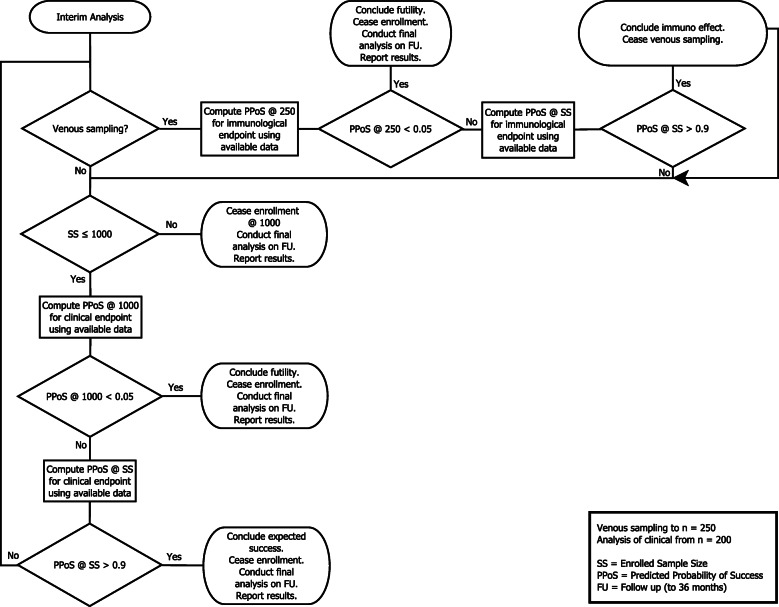
Process flow diagram for interim analyses and decision rules

A simplified flow chart for the interim analyses and decision rules is presented in Fig. 1. The first interim analysis on the immunological endpoint will occur when 70 participants have full (baseline and follow-up) immunologic results. Further interim analyses occur after every subsequent 50 children or after every 3 months, whichever occurs sooner, unless there have been no new entrants. If there are no new blood samples and/or events, then we will defer the full analysis until the next scheduled interim. Analysis of the clinical endpoint will start when 200 children are enrolled in order that there are enough events to meaningfully undertake a time-to-event analysis.

After the maximum number of participants with immunological samples (*n* = 250) has been collected and processed, we will continue to conduct interim analyses every 3 months using the accumulating clinical outcome data until a statistical trigger occurs or the maximum sample size is reached.

The data for assessing the clinical outcomes and/or serious adverse events is obtained from surveillance, which is ongoing until 36 months of age. In brief, each participant’s medical records are checked within 28 to 55 days of vaccination and then every 6 months after vaccination. When medical care cannot be identified, we will make direct contact with the participant’s legally responsible caregiver and/or treating physician to determine whether medical attendance or serious adverse events occurred.

Censoring is complicated by virtue of the intermittent surveillance on each participant; this is especially the case for the interim analyses. At each interim analysis, for participants (1) not yet having had an event, (2) less than 36 months of age and (3) not considered lost to follow-up, we will censor at the time of the last surveillance time. Participants will be censored at 36 months of age if they have not had an event, and if lost to follow-up, censored at the last known time to be in the study. If no surveillance has occurred for an enrolled participant at the time of an interim analysis, then we will not use their data for estimating the posterior.

At each interim analysis up to *n* = 250, we will have incomplete immunological results on the enrolled participants. Additionally, many participants will not have reached the clinical endpoint nor completed follow-up. In order to incorporate all of the available information into the decision framework, we make use of posterior predictive distributions to generate simulated data conditional on our posterior parameter estimates. This process enables us to impute values for subjects with incomplete results [13].

For the immunological outcome, we will compute the joint posterior distribution of the model parameters using methods detailed earlier, namely a logistic regression model with an indicator variable for treatment status. We will use all the available data with complete immunological results.

Next, we will test for futility by computing the predictive probability of observing a treatment effect under the assumption that we continue to the maximum sample size (*n* = 250) for the immunological endpoint. A predictive probability is computed using the following steps.

1. Take a draw from the joint posterior distribution.
2. Use the draw to simulate random variables from a Bernoulli distribution to impute the immunological results that are not yet available for the enrolled participants with pending immunological results and the immunological results for future participants that are currently unenrolled up to the maximum sample size of 250.
3. Combine the observed and the simulated data to form a complete dataset.
4. Fit a logistic regression model to the complete dataset and compute the probability that the treatment effect is above zero.
5. If the probability that the treatment effect is greater than zero is above a threshold value, then consider the trial successful and increment a counter of the number of successful trials.
6. Go back to step 1, repeating the process at least 1000 times.

At the end of the process, we know the number of times that the trial was deemed successful from which we can compute the probability of predicted success (PPoS) as:

6 $$\begin{array}{*{20}l} \textrm{PPoS}= \frac{1}{k} \sum_{1}^{k} \mathrm{I}[\mathbb{P}(\beta_{\text{trt}}>0)>0.97] \end{array} $$

where I() represents an indicator function evaluating to 1 if the contained expression is true and 0 otherwise, *β*_trt_ is the log odds ratio of seroconversion in the treatment arm versus the control arm and *k* is the total number of simulated datasets. If the PPoS is less than the futility threshold, then stop for futility and cease enrolment.

Next, if futility was not established, repeat the above process; however, only impute for the enrolled participants that do not yet have complete immunological data. If the resultant PPoS is greater than the threshold to stop venous sampling, we will cease venous sampling. If either the futility or stop venous sampling thresholds are triggered, we will undertake a final analysis on the immunological outcome once all the enrolled participants (at the time of the trigger) have been followed to completion.

Assuming that the trial has not been stopped for futility, we will start analysing the clinical endpoint at the next scheduled interim analysis after 200 participants have enrolled. The interim analysis process for the clinical outcome is similar to that of the immunological outcome, but with the added complication of censoring.

First, we will compute the joint posterior distribution of the model parameters using methods detailed earlier, namely a Weibull proportional hazards model with an indicator variable for treatment status. To compute the posterior, we will use the data from all participants that have had at least one surveillance visit.

Next, we will test for futility by computing the predictive probability of observing a treatment effect under the assumption that we continue to the maximum sample size (*n* = 1000) for the clinical endpoint. A predictive probability is computed using the following steps.

1. Take a draw from the joint posterior distribution.
2. Use the draw to simulate random variables from a left-truncated Weibull distribution to impute the event times for the enrolled participants that are not yet censored due to age but are yet to have an event. Additionally, use the draw to simulate the event times from a Weibull distribution for the participants not yet enrolled, up to the maximum size (*n* = 1000).
3. If the simulated event times occur after the participant is 36 months old, then censor at 36 months less the age of enrolment, which, if unknown, is drawn from a uniform distribution between 6 and 12 months.
4. Combine the observed and the simulated data to form a complete dataset.
5. Fit a Weibull proportional hazards model to the complete dataset and compute the probability that the treatment effect is below zero.
6. If the probability that the treatment effect is below zero is above a threshold value, then consider the trial successful and increment a counter of the number of successful trials.
7. Go back to step 1, repeating the process at least 1000 times.

At the end of the process, we know the number of times that the trial was deemed successful from which we can compute the probability of predicted success (PPoS) as:

7 $$\begin{array}{*{20}l} \textrm{PPoS}= \frac{1}{k} \sum_{1}^{k} \mathrm{I}[\mathbb{P}(\theta_{\text{trt}}<0)>0.97] \end{array} $$

where I() represents an indicator function evaluating to 1 if the contained expression is true and 0 otherwise, *θ*_trt_ is log hazard ratio for the treatment effect and *k* is the total number of simulated datasets. If the PPoS is less than the futility threshold, then stop for futility and cease enrolment.

Next, if futility was not established, repeat the above process; however, only impute for the enrolled participants that have not yet had an event or have not yet had a visit and are not censored for age. If the imputed event times fall beyond the time of the interim or age to 36 months, then censor appropriately. If the resultant PPoS is greater than the expected success threshold, we will cease the trial for expected success. If either futility or expected success thresholds are triggered, we will undertake a final analysis on the clinical outcome once all the enrolled participants (at the time of the trigger) have been followed to completion.

If none of the above rules are met, we continue enrolling (up to 1000 participants) and venous sampling (up to 250 participants).

If we observe a treatment effect associated with the immunological but none for the clinical outcome, we will conclude that a positive immunological effect has been conferred, but a clinically meaningful benefit has not been demonstrated. If we observe neither immunological nor clinical treatment effects, we will deem the trial to have not met our predefined decision thresholds, but report on the observed probabilities of immunological and clinical treatment effects.

As documented in the protocol [42], the trial may also be stopped at any point at the discretion of the coordinating principal investigator (CPI) or the trial sponsor. The DSMB will advise the CPI according to pre-determined stopping rules or unanticipated safety concerns.

### Safety and adverse events

Two safety and tolerability outcomes are defined in the protocol: the occurrence of intussusception within the first 28 days after randomisation and the occurrence of serious adverse events between randomisation and 36 months. The occurrence of intussusception will be reported in terms of frequency and proportion, by treatment arm and overall. The serious adverse events as defined by [25] will be reported as frequency and proportion, stratified by treatment arm and overall. While no analysis will be performed on the safety and adverse event data, they will be included in the competing risk sensitivity analysis mentioned earlier.

### Pre-specified subgroup analyses

No subgroup analyses were pre-specified in the protocol.

### Sensitivity analyses

As a sensitivity analysis, we will examine the results at the final analysis for the clinical endpoint by fitting a piecewise exponential model that permits the baseline hazard to be a function of treatment, i.e. treatment level time varying hazards [43].

Given that the endpoints are binary and event times (with possible right censoring), there will be no data outliers.

## Data monitoring

The processes around trial monitoring, including data monitoring, are defined in the quality assurance procedures of the ORVAC protocol document.

In brief, data will be sourced from a specifically designed clinical record form (CRF) comprising consent forms, eligibility assessment, visit record, adverse event details and protocol deviations. Data is entered from these sources into a trial database by study personnel. Data queries are raised by the data manager and cleaned by both the data manager and trial statistician. All planned final analyses identified herein are to be performed after the study is completed and the database has been cleaned and locked. However, the interim analyses will be performed on incomplete data in that amendments may occur to the data that was used in an interim at some time after the interim analysis is completed. Our assumption here is that less than 5% of the sample size at any given interim analysis will be subsequently amended.

## Missing data

Within Bayesian analyses, missingness is either ignorable or non-ignorable. In the former case, the parameters relating to the measurement are distinct from those that relate to missingness and the mechanisms are termed either Missing Completely at Random (MCAR) or Missing at Random (MAR). For MCAR, a complete-case analysis will result in reduced efficiency but is unbiased. However, for MAR, a complete-case analysis will be both inefficient and biased. Non-ignorable missingness relates to those data that are Missing Not At Random (MNAR). Under MNAR, an extra model is required to predict the missingness.

One of the advantages of using Bayesian methods is that they offer a natural way to simultaneously impute missing values and fit models on the observed and imputed data [44]. After exploring the amount and patterns of missing data and the association with other variables, we will impute as necessary using a fully Bayesian approach following methods as per [44].

## Evaluation of operating characteristics

Except for the simplest of cases, the operating characteristics for a Bayesian adaptive trial are analytically intractable. Therefore, these are usually estimated by Monte Carlo methods, which are a general purpose tool for optimisation and integration problems [45]. In the context of exploring the operating characteristics of a given trial, the idea is to formulate a data generating process (DGP) that represents expected and plausibly extreme outcomes for the actual trial. However, as is the case here, it may be necessary to simplify the data generating process and/or analysis approach in order to be able to run the simulations within a workable timeframe. Using the assumed DGP, trial data can be simulated many times and the resulting ‘virtual trials’ are analysed using methods described shortly. Various characteristics of the DGP can then be derived. For example, the expected type I error (false positive rate) is derived from data that are generated under a null effect configuration and estimated from the proportion of times that we falsely detect a difference between the treatment arms in the final analysis.

We have examined the operating characteristics of the ORVAC trial in more than 300 scenarios using Monte Carlo simulation of the parameter space associated with a representative data generating, sampling and modelling process. For the immunological endpoint, seroconversion was modelled via a series of independent Bernoulli trials with control arm probabilities of seroconversion (the baseline seroconversion rate) between 0.1 and 0.7 and a change of probability of seroconversion in the treatment arm between 0 (the null case) and 0.15 above the baseline rate. Additionally, we modelled information delays associated with processing the immunological endpoint of 0.5 and 0.7 months. For the clinical endpoint, we modelled time to event as an exponentially distributed random variable. The control arm median time to event was set at 20–50 months and a change in the median time to event varied between 0 (the null case) and 15 months. Event times were censored at 36 months of age. We modelled accrual using a Poisson process generating approximately 30 and 50 participants per quarter. Age at vaccination was modelled using a uniform distribution with lower and upper bounds of 6 and 12 months respectively. The co-primary endpoints were modelled as independent random variables.

For the analyses, we used conjugate prior models because Markov Chain Monte Carlo estimates of the posterior and posterior predictive distributions were prohibitively costly in terms of available CPU resources. Specifically, we used the beta conjugate prior to the binomial likelihood for the immunological endpoint, and the gamma conjugate prior to the exponential likelihood for the clinical endpoint with both sets of priors configured to be weakly informative. We note that while the simulation methods are representative of the analyses we propose for the trial, they are not identical to them.

For each scenario/subset of the parameter space, we simulated 10,000 trials for the null cases and 1000 trials for the trials where each configuration was for a non-zero difference between the treatment arms. We summarised and reviewed the results with other statisticians and study CIs, explored the trial decision probability thresholds and then re-ran the scenarios. The whole process was repeated until acceptable false positive and other trial characteristics were obtained.

For each scenario, all posterior sampling was based on 2000 draws from the relevant distribution. Posterior predictive assessments were based on a further 1000 posterior predictive draws at each interim for both endpoints.

The simulations were written in R and C++, reviewed by JT and stored under version control. The simulations were coded and run by MAJ on Linux-based multi-core servers.

## Probability thresholds for interim and final decisions

Table 2, presented earlier, details the thresholds that were identified through simulation to be used for the interim and final analysis.

## Type I error rate

Table 3 provides examples of the type I error rates obtained over a range of plausible scenarios. In all cases, the type I error/false positive rate is controlled at the *α*<0.05 level for both co-primary endpoints. Adjustment for multiple comparisons is addressed via the probability thresholds that are selected to be used in the analyses.

**Table 3:** Type I error rates for null configurations

Parameters		Accrual per 3 months	Info. delay	Samp. size mean (SD)	Type I error rate	
Median time to event	Prob. Seroconv.				Clinical	Immuno
50	0.7	50	0.7	259 (259.2)	0.036	0.032
50	0.7	30	0.7	247 (238.4)	0.041	0.028
50	0.7	50	0.5	264 (266.3)	0.039	0.030
50	0.7	30	0.5	252 (244.8)	0.043	0.032
50	0.4	50	0.7	261 (262.2)	0.033	0.031
50	0.4	30	0.7	247 (238.6)	0.042	0.029
50	0.4	50	0.5	259 (260.4)	0.037	0.028
50	0.4	30	0.5	248 (242.2)	0.042	0.032
50	0.1	50	0.7	256 (254.9)	0.031	0.031
50	0.1	30	0.7	247 (237.7)	0.038	0.032
50	0.1	50	0.5	259 (259.0)	0.036	0.031
50	0.1	30	0.5	246 (239.2)	0.040	0.032
35	0.7	50	0.7	253 (253.5)	0.034	0.030
35	0.7	30	0.7	245 (236.8)	0.039	0.029
35	0.7	50	0.5	260 (259.0)	0.040	0.030
35	0.7	30	0.5	247 (239.6)	0.043	0.034
35	0.4	50	0.7	264 (263.3)	0.039	0.031
35	0.4	30	0.7	246 (237.6)	0.043	0.031
35	0.4	50	0.5	262 (262.8)	0.038	0.030
35	0.4	30	0.5	247 (237.9)	0.042	0.030
35	0.1	50	0.7	259 (256.7)	0.033	0.031
35	0.1	30	0.7	246 (238.4)	0.035	0.035
35	0.1	50	0.5	259 (258.9)	0.036	0.028
35	0.1	30	0.5	242 (231.9)	0.038	0.030
20	0.7	50	0.7	264 (261.5)	0.037	0.028
20	0.7	30	0.7	244 (233.4)	0.038	0.027
20	0.7	50	0.5	260 (259.1)	0.041	0.029
20	0.7	30	0.5	247 (235.8)	0.043	0.027
20	0.4	50	0.7	258 (256.9)	0.036	0.030
20	0.4	30	0.7	246 (236.9)	0.041	0.029
20	0.4	50	0.5	262 (263.4)	0.040	0.029
20	0.4	30	0.5	248 (237.6)	0.044	0.030
20	0.1	50	0.7	257 (250.7)	0.034	0.031
20	0.1	30	0.7	244 (233.6)	0.035	0.029
20	0.1	50	0.5	256 (254.4)	0.036	0.033
20	0.1	30	0.5	242 (231.1)	0.038	0.032

## Sample size, power and expected success

**Fig. 2:**
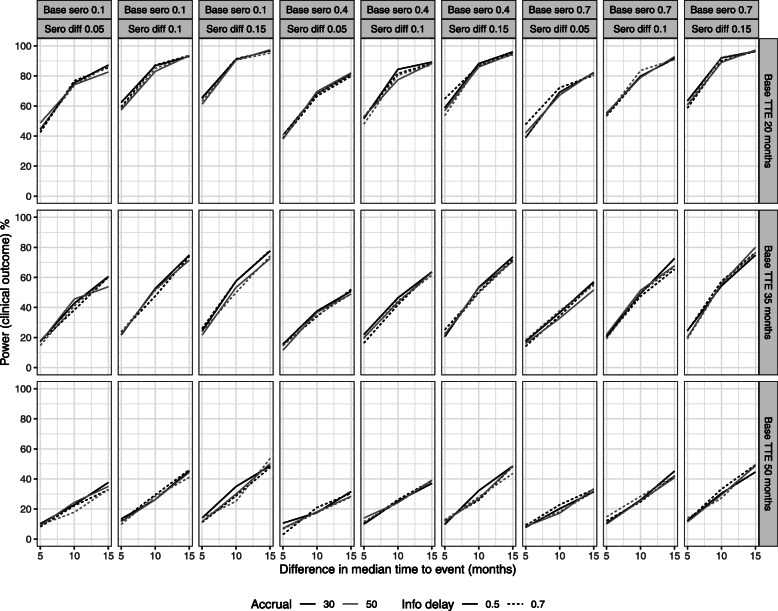
Statistical power for clinical outcome for a range of baseline values, effect sizes and accrual rates

**Fig. 3:**
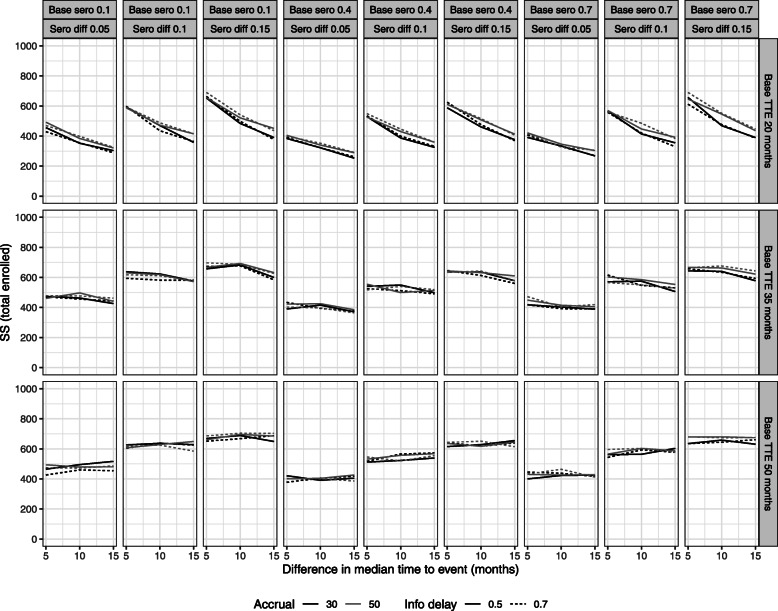
Expected sample size (total enrolled) assessed on clinical and immunological outcome over a range of baseline values, effect sizes and accrual rates

Figures 2 and 3 show the power curves for the clinical outcome and expected total sample size respectively. Other aspects of the operating characteristics including probability of expected success, futility and stopping venous sampling and expected sample size of the immunological endpoint over the parameter space are provided as supplemental material Figs. 4, 5, 6, 7, and 8.

**Fig. 4:**
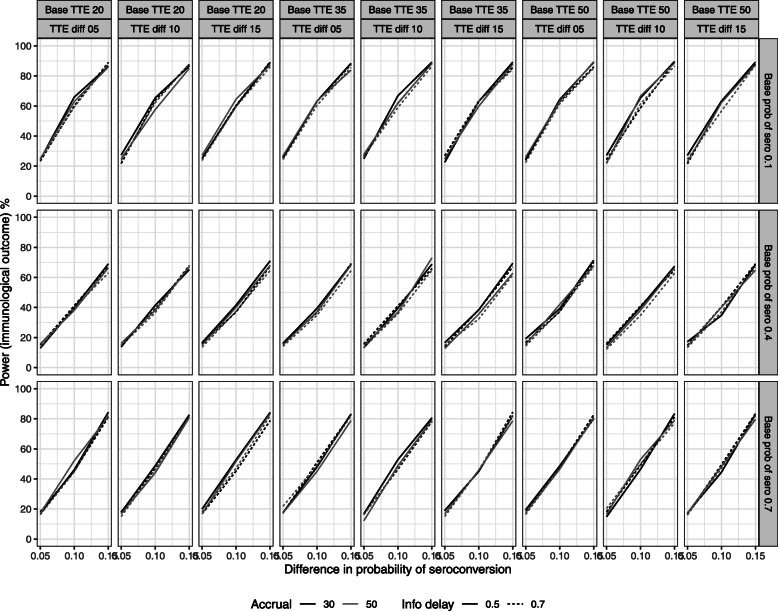
Statistical power for immunological outcome for a range of baseline values, effect sizes and accrual rates

**Fig. 5:**
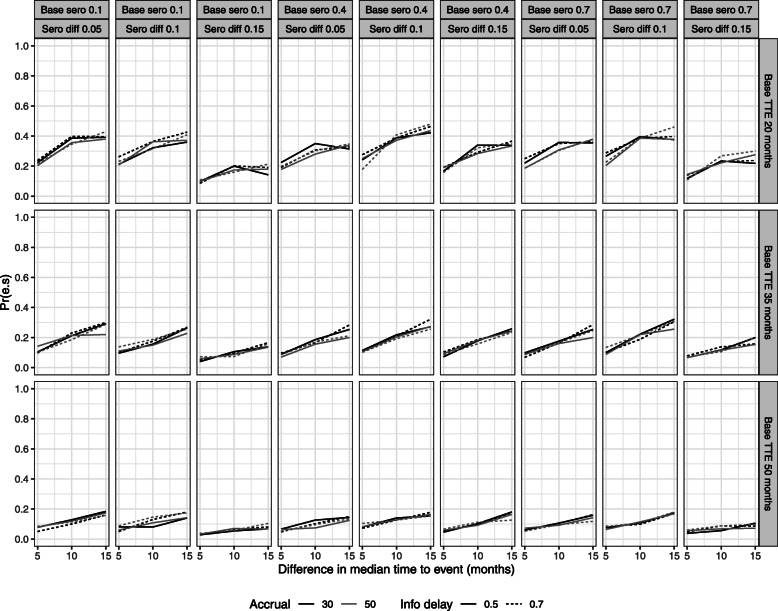
Probability of expected success assessed on clinical outcome over a range of baseline values, effect sizes and accrual rates

**Fig. 6:**
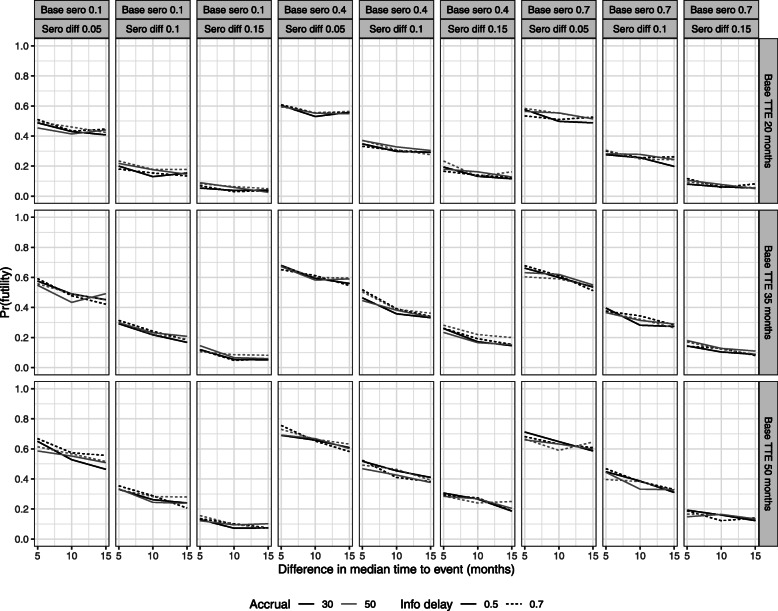
Probability of futility assessed on clinical and immunological outcome over a range of baseline values, effect sizes and accrual rates

**Fig. 7:**
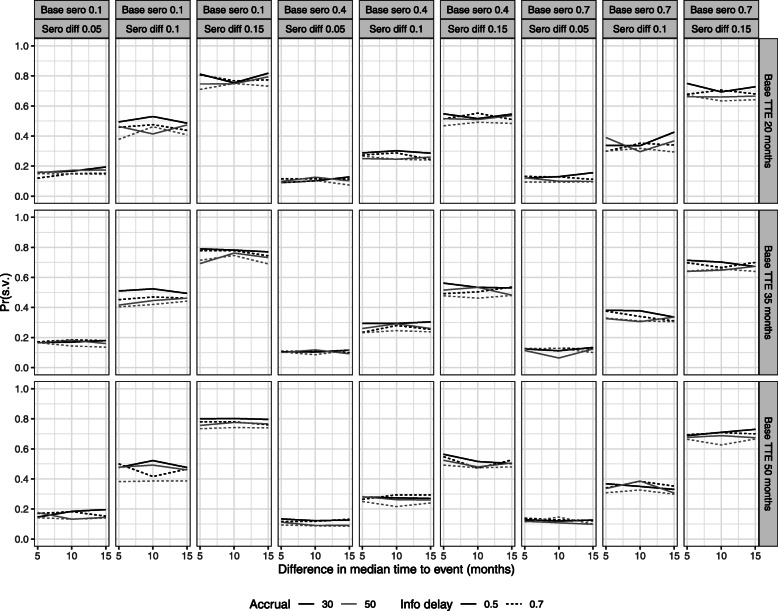
Probability of stopping venous sampling assessed on immunological outcome over a range of baseline values, effect sizes and accrual rates

**Fig. 8:**
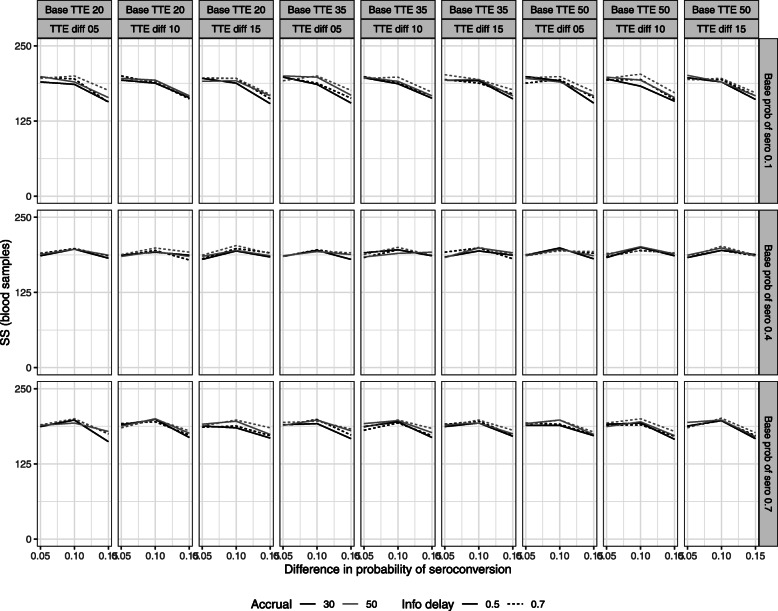
Expected sample size (venous samples) assessed on immunological outcome over a range of baseline values, effect sizes and accrual rates

The clinical and immunological outcome are analysed independently. For the clinical outcome, power increases monotonically as a function of increasing effect size and lower baseline median time-to-event scenarios. For a baseline median time to event of 35 months, power climbs from 50 to 75% as the difference between the median time to event in the two arms increases from 10 to 15 months. At a baseline time to event of 20 months, the power to detect a 10-month difference is over 70%. Power was relatively insensitive to the accrual rates and information delays that we examined.

For the immunological outcome, power is largely independent of the clinical outcome configuration. For a baseline probability of seroconversion of 0.4, while the power to detect a 0.1 difference in probability of seroconversion is only around 40%, this increases to around 70% to detect a difference of 0.15. However, when the baseline probability of seroconversion is 0.1, the power to detect a 0.1 difference is in excess of 60% and to detect a 0.15 difference is well in excess of 80%.

The probability of expected success increases as a function of the difference between the median time to event in the treatment versus control arm. When the difference between the probability of seroconversion between the treatment and placebo arms is 0.05, the probability of expected success is only around 0.2. However, as the difference in the seroconversion probabilities increases, the probability of expected success becomes increasingly likely.

The probability of stopping for futility is dependent on both the clinical and immunological outcomes. For a difference between the median time to event equal to 5 months, the probability of futility is up to 0.7. However, as the difference between the median time to event and probability increases, the probability of futility drops to less than 0.1.

The probability of stopping venous sampling before reaching the maximum of *n* = 250 is only around 0.2 when the difference in the probability of seroconversion between the two arms is 0.05. However, this increases rapidly as the difference between the groups increases and is up to 0.8 when the difference in the probability in the two arms is 0.15.

The expected sample size reduces as a function of clinical endpoint effect size but increases as a function of the immunological endpoint effect size. This is because the probability of ceasing the trial for futility remains high (in excess of 50%) when the immunological endpoint effect size is low. However, as the immunological endpoint effect size increases, so does the probability that the trial will continue to accumulate enough data to detect a clinical treatment effect. Based on the scenarios we simulated, we anticipate a minimum expected sample size of around 300 participants and up to a maximum sample size of 700. We anticipate that the expected number of blood samples will range from 150 to 200.

## Analysis software

We used R version 3.5.3, RStudio version 1.1.423 and C++ for the trial simulations and will use these or later versions in the trial analyses.

## Discussion

The motivations, context and procedures for implementing the ORVAC trial have been described in the protocol. In this document, we have provided a detailed specification of the statistical matters and decision processes relating to the interim and final analyses. We discussed the Monte Carlo simulations of plausible and extreme scenarios used to establish the operating characteristics of the ORVAC trial.

As called for in comparable trials investigating alternative dosing schedules [46, 47] and proposed frameworks for evaluating rotavirus vaccines [48], ORVAC implements co-primary immunological and clinical endpoints. This feature enables us to examine the extent to which laboratory findings translate into a public health benefits. The adaptive sample-size design with regular interim analyses enables us to stop the trial for futility (due to unacceptably low chance of observing a treatment effect) or expected success (due to overwhelming evidence of a treatment effect). These adaptive elements have been shown to decrease unnecessary expense of resources, reduce risk to participants and minimise the chance of inconclusive results [10]. The results from the analyses documented herein will be published in peer-reviewed literature. At a minimum, these publications will include independent reporting on the final immunological outcome and the final clinical outcome after the relevant stopping rules have been triggered and the follow-up period completed.

### COVID-19 pandemic

The COVID-19 pandemic has impacted the ORVAC trial due to policy responses as follows: (1) permits for travel to remote Top End communities were revoked by Northern Land Council, (2) research has been postponed in all Congress sites by the Central Australian Aboriginal Congres and (3) Menzies Institute suspended all research involving direct contact between researchers and participants

In response, the ORVAC team suspended recruitment and day 28–55 follow-up visits as of 23 March 2020 for all sites. However, medical record review will continue as per the protocol. All blood samples have been sent from the Darwin and Alice laboratories to Perth, deep frozen and will be processed once the Perth pathology laboratory reopens. As the first participant was enrolled into ORVAC on 27 March 2018, we define the pre-COVID-19 time period as *27 March 2018 to 23 March 2020*. As of 5 June 2020, the Darwin sites have reopened for enrollment. Further discussion of COVID-19 impacts have been included in the supplementary documents (Additional file 2).

## Supplementary information

Supplementary information accompanies this paper at

**Additional file 1** SPIRIT 2013 Checklist. Standard Protocol Items: Recommendations for Interventional Trials.

**Additional file 2** Impacts relating to COVID-19 pandemic (ORVAC Trial). Documentation on potential impacts associated with the COVID-19 pandemic on the ORVAC Trial.

## Data Availability

The investigators will undertake to make patient-level data available for independent analysis subject to any requisite approval from the relevant ethics and governance committees.
