# Supplementary material for: The ORVAC trial: a phase IV, double-blind, randomised, placebo-controlled clinical trial of a third scheduled dose of Rotarix rotavirus vaccine in Australian Indigenous infants to improve protection against gastroenteritis: a statistical analysis plan
Source: Trials. 2020 Aug 26;21:741. doi: 10.1186/s13063-020-04602-w (PMC7447587; doi:10.1186/s13063-020-04602-w)
Supplement: Supplementary file 1 — Additional file 1 SPIRIT 2013 Checklist. Standard Protocol Items: Recommendations for Interventional Trials. [file 13063_2020_4602_MOESM1_ESM.docx]

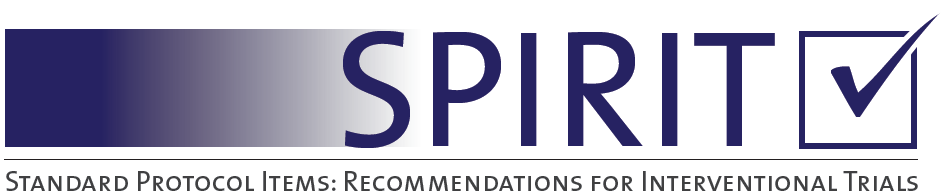


SPIRIT 2013 Checklist: Recommended items to address in a clinical trial protocol and related documents*

| Section/item | Item No | Description | Addressed on page number |
| --- | --- | --- | --- |
| **Administrative information** | | |  |
| Title | 1 | Descriptive title identifying the study design, population, interventions, and, if applicable, trial acronym | protocol manuscript page 1, sap page 1 |
| Trial registration | 2a | Trial identifier and registry name. If not yet registered, name of intended registry | protocol manuscript page 5, sap page 2 |
|  | 2b | All items from the World Health Organization Trial Registration Data Set | clinicaltrials.gov, NCT02941107 |
| Protocol version | 3 | Date and version identifier | The current protocol is version 6 dated 19 March 2019.  The protocol will be submitted for publication with BMJ.  The current statistical analysis plan is numbered version 4, dated 8 June 2019 and will be submitted to Trials |
| Funding | 4 | Sources and types of financial, material, and other support | Included in the protocol manuscript page 25 & 26 |
| Roles and responsibilities | 5a | Names, affiliations, and roles of protocol contributors | protocol manuscript page 1 & 2, sap page 1, 17 |
|  | 5b | Name and contact information for the trial sponsor | name included in protocol page 18, sap page 19 |
|  | 5c | Role of study sponsor and funders, if any, in study design; collection, management, analysis, and interpretation of data; writing of the report; and the decision to submit the report for publication, including whether they will have ultimate authority over any of these activities | Included in the protocol manuscript page 18 |
|  | 5d | Composition, roles, and responsibilities of the coordinating centre, steering committee, endpoint adjudication committee, data management team, and other individuals or groups overseeing the trial, if applicable (see Item 21a for data monitoring committee) | Included in the protocol manuscript page 18. |
| Introduction |  |  |  |
| Background and rationale | 6a | Description of research question and justification for undertaking the trial, including summary of relevant studies (published and unpublished) examining benefits and harms for each intervention | protocol manuscript page 7, sap page 2 |
|  | 6b | Explanation for choice of comparators | protocol manuscript page 10, sap page 2, 6 |
| Objectives | 7 | Specific objectives or hypotheses | protocol manuscript page 9, sap page 5 |
| Trial design | 8 | Description of trial design including type of trial (eg, parallel group, crossover, factorial, single group), allocation ratio, and framework (eg, superiority, equivalence, noninferiority, exploratory) | protocol manuscript page 9, sap page 4 |
| Methods: Participants, interventions, and outcomes | | |  |
| Study setting | 9 | Description of study settings (eg, community clinic, academic hospital) and list of countries where data will be collected. Reference to where list of study sites can be obtained | protocol manuscript page 9, sap page 5 |
| Eligibility criteria | 10 | Inclusion and exclusion criteria for participants. If applicable, eligibility criteria for study centres and individuals who will perform the interventions (eg, surgeons, psychotherapists) | protocol manuscript page 10, (more in main protocol document page 24) |
| Interventions | 11a | Interventions for each group with sufficient detail to allow replication, including how and when they will be administered | protocol manuscript page 13, sap page 2, 3 |
|  | 11b | Criteria for discontinuing or modifying allocated interventions for a given trial participant (eg, drug dose change in response to harms, participant request, or improving/worsening disease) | protocol manuscript page 15, sap page 6 |
|  | 11c | Strategies to improve adherence to intervention protocols, and any procedures for monitoring adherence (eg, drug tablet return, laboratory tests) | The intervention is delivered once-off at the time of enrolment by study staff so there are no strategies required for treatment adherence. |
|  | 11d | Relevant concomitant care and interventions that are permitted or prohibited during the trial | Relevant exclusion criteria are covered in the protocol manuscript page 12 |
| Outcomes | 12 | Primary, secondary, and other outcomes, including the specific measurement variable (eg, systolic blood pressure), analysis metric (eg, change from baseline, final value, time to event), method of aggregation (eg, median, proportion), and time point for each outcome. Explanation of the clinical relevance of chosen efficacy and harm outcomes is strongly recommended | protocol manuscript page 10 |
| Participant timeline | 13 | Time schedule of enrolment, interventions (including any run-ins and washouts), assessments, and visits for participants. A schematic diagram is highly recommended (see Figure) | protocol manuscript page 9 references relevant fig and tab, (see page 23 & 24 for figure/ table) |
| Sample size | 14 | Estimated number of participants needed to achieve study objectives and how it was determined, including clinical and statistical assumptions supporting any sample size calculations | protocol manuscript page 16 & 17, sap page 4, 14 |
| Recruitment | 15 | Strategies for achieving adequate participant enrolment to reach target sample size | main protocol page 14; protocol manuscript page 10 |
| **Methods: Assignment of interventions (for controlled trials)** | | |  |
| Allocation: |  |  |  |
| Sequence generation | 16a | Method of generating the allocation sequence (eg, computer-generated random numbers), and list of any factors for stratification. To reduce predictability of a random sequence, details of any planned restriction (eg, blocking) should be provided in a separate document that is unavailable to those who enrol participants or assign interventions | protocol manuscript page 13, sap page 5 |
| Allocation concealment mechanism | 16b | Mechanism of implementing the allocation sequence (eg, central telephone; sequentially numbered, opaque, sealed envelopes), describing any steps to conceal the sequence until interventions are assigned | protocol manuscript page 13 |
| Implementation | 16c | Who will generate the allocation sequence, who will enrol participants, and who will assign participants to interventions | protocol manuscript page 13 |
| Blinding (masking) | 17a | Who will be blinded after assignment to interventions (eg, trial participants, care providers, outcome assessors, data analysts), and how | protocol manuscript page 13, sap page 5 |
|  | 17b | If blinded, circumstances under which unblinding is permissible, and procedure for revealing a participant’s allocated intervention during the trial | sap page 5, also main protocol page 24 |
| **Methods: Data collection, management, and analysis** | | |  |
| Data collection methods | 18a | Plans for assessment and collection of outcome, baseline, and other trial data, including any related processes to promote data quality (eg, duplicate measurements, training of assessors) and a description of study instruments (eg, questionnaires, laboratory tests) along with their reliability and validity, if known. Reference to where data collection forms can be found, if not in the protocol | protocol manuscript page 14, main protocol page 37 |
|  | 18b | Plans to promote participant retention and complete follow-up, including list of any outcome data to be collected for participants who discontinue or deviate from intervention protocols | protocol manuscript page 14,15 , main protocol page 21 |
| Data management | 19 | Plans for data entry, coding, security, and storage, including any related processes to promote data quality (eg, double data entry; range checks for data values). Reference to where details of data management procedures can be found, if not in the protocol | protocol manuscript page 14, main protocol page 37 |
| Statistical methods | 20a | Statistical methods for analysing primary and secondary outcomes. Reference to where other details of the statistical analysis plan can be found, if not in the protocol | protocol manuscript page 15-18, sap page 7-9 |
|  | 20b | Methods for any additional analyses (eg, subgroup and adjusted analyses) | protocol manuscript page 15-18, sap page 9-13 |
|  | 20c | Definition of analysis population relating to protocol non-adherence (eg, as randomised analysis), and any statistical methods to handle missing data (eg, multiple imputation) | sap page 5, 14 |
| **Methods: Monitoring** | | |  |
| Data monitoring | 21a | Composition of data monitoring committee (DMC); summary of its role and reporting structure; statement of whether it is independent from the sponsor and competing interests; and reference to where further details about its charter can be found, if not in the protocol. Alternatively, an explanation of why a DMC is not needed | Protocol manuscript page 19 |
|  | 21b | Description of any interim analyses and stopping guidelines, including who will have access to these interim results and make the final decision to terminate the trial | sap page 13 |
| Harms | 22 | Plans for collecting, assessing, reporting, and managing solicited and spontaneously reported adverse events and other unintended effects of trial interventions or trial conduct | protocol manuscript page 18, main protocol page 37 |
| Auditing | 23 | Frequency and procedures for auditing trial conduct, if any, and whether the process will be independent from investigators and the sponsor | what to say? |
| Ethics and dissemination | | |  |
| Research ethics approval | 24 | Plans for seeking research ethics committee/institutional review board (REC/IRB) approval | protocol manuscript page 5, main protocol page 39 |
| Protocol amendments | 25 | Plans for communicating important protocol modifications (eg, changes to eligibility criteria, outcomes, analyses) to relevant parties (eg, investigators, REC/IRBs, trial participants, trial registries, journals, regulators) | protocol manuscript page 5 |
| Consent or assent | 26a | Who will obtain informed consent or assent from potential trial participants or authorised surrogates, and how (see Item 32) | protocol manuscript page 11. main protocol page 19 |
|  | 26b | Additional consent provisions for collection and use of participant data and biological specimens in ancillary studies, if applicable | main protocol page 14, 19 |
| Confidentiality | 27 | How personal information about potential and enrolled participants will be collected, shared, and maintained in order to protect confidentiality before, during, and after the trial | protocol manuscript page 11, 14, main protocol page 37 |
| Declaration of interests | 28 | Financial and other competing interests for principal investigators for the overall trial and each study site | protocol manuscript page 26, sap page 17 |
| Access to data | 29 | Statement of who will have access to the final trial dataset, and disclosure of contractual agreements that limit such access for investigators | Protocol manuscript page 18. Main protocol page 39 |
| Ancillary and post-trial care | 30 | Provisions, if any, for ancillary and post-trial care, and for compensation to those who suffer harm from trial participation | main protocol page 40 |
| Dissemination policy | 31a | Plans for investigators and sponsor to communicate trial results to participants, healthcare professionals, the public, and other relevant groups (eg, via publication, reporting in results databases, or other data sharing arrangements), including any publication restrictions | protocol manuscript page 5, sap page 17 |
|  | 31b | Authorship eligibility guidelines and any intended use of professional writers | main protocol page 40 |
|  | 31c | Plans, if any, for granting public access to the full protocol, participant-level dataset, and statistical code | aim to publish manuscript in BMJ |
| Appendices |  |  |  |
| Informed consent materials | 32 | Model consent form and other related documentation given to participants and authorised surrogates | protocol manuscript page 11 |
| Biological specimens | 33 | Plans for collection, laboratory evaluation, and storage of biological specimens for genetic or molecular analysis in the current trial and for future use in ancillary studies, if applicable | main protocol, Section 10, page 25-26. |

*It is strongly recommended that this checklist be read in conjunction with the SPIRIT 2013 Explanation & Elaboration for important clarification on the items. Amendments to the protocol should be tracked and dated. The SPIRIT checklist is copyrighted by the SPIRIT Group under the Creative Commons “[Attribution-NonCommercial-NoDerivs 3.0 Unported](http://www.creativecommons.org/licenses/by-nc-nd/3.0/)” license.
