# Supplementary material for: The ORVAC trial: a phase IV, double-blind, randomised, placebo-controlled clinical trial of a third scheduled dose of Rotarix rotavirus vaccine in Australian Indigenous infants to improve protection against gastroenteritis: a statistical analysis plan
Source: Trials. 2020 Aug 26;21:741. doi: 10.1186/s13063-020-04602-w (PMC7447587; doi:10.1186/s13063-020-04602-w)
Supplement: Supplementary file 2 — Additional file 2 Impacts relating to COVID-19 pandemic (ORVAC Trial). Documentation on potential impacts associated with the COVID-19 pandemic on the ORVAC Trial. [file 13063_2020_4602_MOESM2_ESM.pdf]

Considerations relating to the impacts from the COVID-19 pandemic on the ORVAC Trial

Additional file 2

Mark Jones
Wesfarmers Centre of Vaccines and Infectious Diseases
Telethon Kids Institute

June 9, 2020

1 Overview

The ORVAC trial investigates the addition of a third scheduled dose of RV vaccine in an attempt to offer greater protection against childhood AGE in aboriginal infants. On entry into the study, baseline data is recorded, and a blood sample is taken. On the same day, the nurse administers the IMP/placebo. Between day 14-21 an (online) medical review is undertaken and then between day 28 to 55 a follow up blood sample is taken. The child is then followed up (remotely) at 6 month intervals until age 36 months to determine when they attend hospital for diarrhoea/gastroenteritis.

Recruitment, IMP/placebo administration and day 28-55 follow up (for second blood sample) require direct human interaction. These are the aspects of the study that are primarily impacted by the restrictions associated with the COVID-19 pandemic.

As of 23 Mar 2020 (the date the study was suspended) we have 203 enrolments, 201 baseline and 137 follow up blood samples. The maximum sample size for blood samples is specified to be 250.

This (living) document captures the exercise of thinking and responding to impacts on the ORVAC trial (primarily relating to statistical matters) that arise out of the covid-19 pandemic.

2 Blinding

Unblinding might be necessary in studies where the intervention is provided over a prolonged period of time and access to the participant was no longer possible. In ORVAC the IMP/placebo is administered on day 1 of the study. Therefore, it does not appear to be necessary for ORVAC to undergo any unblinding.

3 Treatment adherence

As IMP/placebo is administered on day 1 of the study, adherence is not an issue.

4 Reopening trial/recruitment

The date when the trial be fully reopened is unknown and the situation changes daily. As of 5 Jun 2020, the Darwin site reopened. If that is indiciative of national policies then we anticipate that the other sites will reopen within the next month or so. However, we note the possibility of being impacted by successive waves of the virus. If this latter scenario occurs, then we could see sites reopen, only to be closed again due to cases of COVID-19 re-escalating. This latter situation is perhaps improbable, but nevertheless is worth mentioning to keep it in mind. Clearly, the suspension impacts recruitment and this will likely impact the trial operating characteristics such as statistical power.

5 Protocol deviations

We will be unable to collect the second blood samples in 15 participants in the specified follow up window. This constitutes a protocol deviation as it deviates from study procedures and impacts the integrity of the data in that it increases missingness. We will report the deviations, noting the exceptional situation in which they arose. The particular form of missingness in this situation is known as missing at random (an indicator variable for covid-19 would predict this missingness perfectly). MAR is one of the two types of ignorable missingness in which multiple imputation can be applied.

6 Potential bias/confounding

Randomisation helps minimise confounding (at baseline); confounding being a difference between treatment groups in terms of participant characteristics (that are not on the causal pathway) that impact the association between the treatment and outcome, e.g. age, demographics etc. that lead to spurious conclusions. If the groups are balanced at the start, then one hopes that the difference at the end of the trial can be reasonably attributed to the intervention (so long as we do not have LTFU that leads to imbalance).

Social habits have been disrupted by the COVID-19 pandemic. Social distancing measures along with travel restrictions have been implemented and enforced to varying degrees across Australia. Increased hand hygiene has been widely promoted. While conceivably, these factors have the potential to impact on the incidence of diarrhoea (a necessary precursor to confounds), the occurrence of the pandemic is equally distributed across the treatment groups and it is unlikely although possible that LTFU might increase. Therefore, we think confounding is unlikely to impact the study.

Another consideration relates to the potential for an increase in parental apprehension regarding visiting hospitals and medical centres during the COVID-19 pandemic. This could reduce the probability of medical attendance, although again, non-differentially across arms.

7 Exclusions

We do not intend to exclude participants with COVID-19 from the trial.

8 Funding

As it is unknown when recruitment will be able to restart, we will defer the decision as to whether to seek a NHMRC grant extension. If necessary we will reassess in Oct 2020 and decide on this then.

9 Trial operating characteristics

By definition, the accrual rate has now dropped to zero and we do not know when the trial will be restarted. This could have an impact on the trial (frequentist) operating characteristics such as statistical power and we plan to re-run the simulations under revised accrual rates.

10 Interim analyses

The next interim analysis is the fourth, current in progress. While we cannot include the pending blood sample results (mentioned earlier) we will provide a detailed summary of the trial status and analyses the first set of data for the clinical endpoint (which is specified to start from 200 enrolees).

We envisage that by the interim after next (3 months hence) there is a chance that the Perth labs will be reopened. If this occurs, we will be able to get the pending blood samples processed and undertake the analysis of the remaining bloods at that time. The stopping rules will remain as they are currently specified.

After this (and assuming the trial has not triggered a stopping rule) we have the situation of the remaining 47 blood samples. As noted, it is unclear when sites will be reopening, to what extent contact procedures might change and how quickly we will be able to get enrolments up to speed again. So, it is unclear as to when the next enrolments (and thus blood samples) will be collected. Moreover, subsequent follow up bloods (on which the analyses are based) will not begin until one or two months after the first enrolment.

We will proceed with the study as planned. That is, every 3 months a new interim will occur using the current time to event and available blood sample data. In the event that we think that sites may be opened but there is some concern that they would close again prior to us being able to get the second blood sample, then we will need to make a decision on the ethics of taking a first blood sample. The interim analyses and decision logic will not be modified for the COVID-19 period. A section of the interim report will be devoted to updates relating to the COVID-19 impacts. Additional to baseline characteristics, we will report numbers of COVID-19 confirmed/unconfirmed cases if applicable.

11 Final analyses

Consideration is being given to how or if to modify the final analysis to adjust for the impacts either by covid-19 disease or the social movement and other life as usual restrictions.

As many other trials are no doubt doing, we will incorporate a (time dependent for the survival analysis) covariate to adjust for the COVID-19 period into our analyses. Rather than make changes to the primary analyses, we will do this in the context of a sensitivity analysis.

Large numbers of COVID-19 related deaths are not considered likely for this study cohort. Furthermore, providing separate analyses of the COVID-19 cases is likely to provide little value.
